# Antiproliferative Effects and Mechanisms of Liver X Receptor Ligands in Pancreatic Ductal Adenocarcinoma Cells

**DOI:** 10.1371/journal.pone.0106289

**Published:** 2014-09-03

**Authors:** Nicholes R. Candelaria, Sridevi Addanki, Jine Zheng, Trang Nguyen-Vu, Husna Karaboga, Prasenjit Dey, Chiara Gabbi, Lise-Lotte Vedin, Ka Liu, Wanfu Wu, Philip K. Jonsson, Jean Z. Lin, Fei Su, Lakshmi Reddy Bollu, Sally E. Hodges, Amy L. McElhany, Mehdi A. Issazadeh, William E. Fisher, Michael M. Ittmann, Knut R. Steffensen, Jan-Åke Gustafsson, Chin-Yo Lin

**Affiliations:** 1 Center for Nuclear Receptors and Cell Signaling, Department of Biology and Biochemistry, University of Houston, Houston, Texas, United States of America; 2 Division of Clinical Chemistry, Department of Laboratory Medicine, Karolinska Institutet, Karolinska University Hospital Huddinge, Stockholm, Sweden; 3 Department of Biosciences and Nutrition at NOVUM, Karolinska Institutet, Huddinge, Sweden; 4 Center for Diabetes Research, Houston Methodist Research Institute, Houston, Texas, United States of America; 5 Michael E. DeBakey Department of Surgery, Baylor College of Medicine, Houston, Texas, United States of America; 6 The Elkins Pancreas Center at Baylor College of Medicine, Houston, Texas, United States of America; 7 Department of Pathology and Immunology, Baylor College of Medicine, Houston, Texas, United States of America; University of Barcelona, Spain

## Abstract

Pancreatic ductal adenocarcinoma (PDAC) is difficult to detect early and is often resistant to standard chemotherapeutic options, contributing to extremely poor disease outcomes. Members of the nuclear receptor superfamily carry out essential biological functions such as hormone signaling and are successfully targeted in the treatment of endocrine-related malignancies. Liver X receptors (LXRs) are nuclear receptors that regulate cholesterol homeostasis, lipid metabolism, and inflammation, and LXR agonists have been developed to regulate LXR function in these processes. Intriguingly, these compounds also exhibit antiproliferative activity in diverse types of cancer cells. In this study, LXR agonist treatments disrupted proliferation, cell-cycle progression, and colony-formation of PDAC cells. At the molecular level, treatments downregulated expression of proteins involved in cell cycle progression and growth factor signaling. Microarray experiments further revealed changes in expression profiles of multiple gene networks involved in biological processes and pathways essential for cell growth and proliferation following LXR activation. These results establish the antiproliferative effects of LXR agonists and potential mechanisms of action in PDAC cells and provide evidence for their potential application in the prevention and treatment of PDAC.

## Introduction

Pancreatic ductal adenocarcinoma (PDAC) is among the most deadly cancers, with a combined (all four stages) survival rate of 5% after five years [1]. Localized neoplasms represent about 20% of diagnosed cases and are resected using the Whipple procedure [2]. PDAC is often asymptomatic until the disease is late in its progression and tends to be poorly vascularized and resistant to the standard-of-care chemotherapeutic agent gemcitabine, a cytidine nucleoside analog that blocks DNA replication [3]. Gemcitabine improves median survival by just over one month when compared to 5-fluorouracil [4]. Recent advances in PDAC treatment pairs gemcitabine with EGFR inhibitors, such as erlotinib or cetuximab, and this combination improved median survival by less than two weeks [5], [6]. Alternative strategies are clearly needed to improve survival and quality of life for PDAC patients.

Members of the nuclear receptor (NR) superfamily of ligand-dependent transcription factors carry out vital cellular functions and are highly druggable targets [7]. NRs are modulated by steroidal and non-steroidal compounds in maintenance of normal metabolism, development, and immune responses [8], [9]. Because NRs have ligand-binding domains with highly specific binding pockets, they can be targeted by a plethora of natural and synthetic compounds in the treatment of autoimmunity, diabetes, and hormone-dependent malignancies of the breast and prostate [8], [9]. For example, estrogen receptor plays a key role in breast cancer and is targeted by selective estrogen receptor modulators (SERMS) in the prevention and treatment of hormone-dependent breast cancers [10]. The androgen receptor is similarly targeted in the treatment of prostate cancers.

Liver X receptors (LXRs) are members of the nuclear receptor superfamily and have been studied extensively for their roles in regulating cholesterol, glucose, fatty acid metabolism, and inflammatory related pathways [8]. Two isoforms have been described, LXRα and LXRβ, that despite common characteristics (high sequence homology, heterodimerization with 9-cis retinoic acid receptors, and a similar ligand profile) have distinct and specific functions [11]. LXRs are activated by a variety of endogenous ligands in normal homeostasis (27-hydroxycholesterol, 20(S)-hydroxycholesterol), or by synthetic ligands such as GW3965 or T0901317 that were developed for the treatment of atherosclerosis. Recent studies in rodents have shown that LXRβ is strongly expressed in pancreatic ductal epithelial cells and LXRβ−/− mice develop a severe pancreatic exocrine insufficiency [12]. However, it is not know whether LXRβ or its ligand may affect normal exocrine pancreatic function or the development of malignancies in humans. Studies of LXR ligands in colon, breast, prostate, lung, and skin cancer cells indicate a potential role for these ligands and LXRs in cancer cell proliferation [13]. Treatment of LNCaP prostatic cells with LXR agonists suppressed their growth in xenograft models [14]. LXR agonists are also antiproliferative in breast cancer cell lines by disrupting both estrogen-dependent proliferation and cell cycle machinery [15], [16]. In addition, female mice lacking LXRβ spontaneously undergo a process of gallbladder carcinogenesis suggesting a specific role of this receptor in regulating cell proliferation [17]. Interestingly, the antiproliferative effect of LXR ligands is potentiated by treatment with 9-cis-retinoic acid in pancreatic islet cells [18]. Based on these observations, we hypothesized that LXR ligands may block cancer cell growth in PDAC. In this study, we examined the effects of LXR agonists on PDAC cells and identified potential mechanisms of action.

## Materials and Methods

### Ethical Statement

De-identified human samples utilized in the study were obtained from the Texas Cancer Research Biobank (http://txcrb.org/index.html) that collected the samples following patient consent and collection protocol (H-29198) approved by the Baylor College of Medicine Institutional Review Board. The use of the tissues by the authors was exempt from institutional review as confirmed by the University of Houston Institutional Review Board.

### Immunohistochemistry

Representative sections (n = 8) of pancreatic adenocarcinoma were obtained from Texas Cancer Research Biobank. 4 males and 4 females were studied (age 40–69). Sections were dewaxed in xylene and rehydrated through graded ethanol. After antigen retrieval with PT module (Thermo Scientific) for 17 minutes at 97°C, sections were incubated in 3% H_2_O_2_ in 50% methanol for 30 min at room temperature to quench endogenous peroxidase. To block nonspecific binding, sections were incubated in PBS containing 1% BSA and 0.1% Nonidet P-40 for 1 h at room temperature. Primary antibody reactions were incubated at 4°C overnight. Goat anti-LXRβ and anti-LXRα antibodies were developed as previously described [12], [19] and used at 1∶50 dilution in 1% BSA and 0.1% Nonidet P-40. Negative controls were incubated with PBS containing 1% BSA and 0.1% Nonidet P-40 without primary antibody. After washing, sections were incubated with goat-probe (Biocare Medical, GHP516) for 15 minutes, then washed in PBS and incubated with goat-on-rodent-HRP polymer (Biocare Medical, GHP516) for 15 minutes. After washing in PBS, sections were developed with 3,3′-diaminobenzidine tetrahydrochloride substrate (DAKO) and then counterstained with Mayer's hematoxylin. Sections were dehydrated through a graded ethanol series and xylene and finally mounted.

### Cell Lines and Tissue Culture

Three human pancreatic cancer cell lines were selected for these studies, BxPC-3, MIA-PaCa-2, PANC-1, (American Type Culture Collection, Rockville, MD, USA). MIA-PaCa-2 and PANC-1 were grown in Dulbecco's modified Eagle's medium (Invitrogen, Carlsbad, CA, USA) containing high Glucose with HEPES and supplemented with 10% fetal bovine serum. BxPC3 cells were cultured in DMEM F-12 (Invitrogen), containing HEPES and Glutamine and supplemented with 10% FBS (Hyclone, Logan, UT, USA).

### Cell Treatments, Gene Knockdowns, and Cell Proliferation Assays

Cells were treated with GW3965 (Tocris Bioscience, Bristol, UK), T0901317 (Tocris Bioscience, Bristol, UK), gemcitabine (Sigma-Aldrich, St. Louis, MO, USA) at indicated concentrations or ethanol as a vehicle. Cell proliferation was measured by MTS metabolic rate assays using CellTiter96 AQueous One Solution (Promega, Madison, WI, USA) following manufacturer's protocol or standard trypan blue exclusion assays using the Countess automated cell counter (Invitrogen) or hemocytometer. Statistical analysis of assay results was performed using the two-tailed Student's t-test. Experiments were performed in triplicate. LXR knockdown experiments were performed by transfecting PDAC cells with pooled targeting siRNA against LXRα and LXRβ following manufacturer's (Thermo Scientific Dharmacon, Lafayette, CO, USA) protocol. Transfections with scrambled siRNA were included as negative controls.

### Cell Cycle Analysis and BrdU Incorporation Assays

Cells were treated with 10 µM GW3965 for 72 hours and then pulsed with 10 µM BrdU for 1 hour. Treated cells were then trypsinized and fixed in 70% ethanol and stored at −20°C for 24 hours. DNA was denatured in 2 M HCl/0.5% Triton-X and then neutralized in 100 mM sodium borate. FITC-conjugated anti-BrdU antibody was then added to bind incorporated BrdU. Fixed cells were incubated at 37°C for 30 minutes with 50 µg/ml of propidium iodide and 10 µg/ml RNase A. FACS Aria 111 Cell Sorter (BD Biosciences) utilized for data collection, and the data were analyzed using FlowJo software program.

### Clonogenic Assay

Cells were seeded in 100 mm plates and treated with LXR ligand for one week (MIA-PaCa-2) or two weeks (BxPC-3 and Panc-1). At the end of treatment period, cells were washed with PBS and fixed in 4% formaldehyde and washed again with PBS. Colonies were then stained with crystal violet (Sigma-Aldrich), scanned, and quantified using the Clono-Counter software [20].

### Microarray and Data Analysis

Total RNA from each cell-line was isolated using RNeasy columns (Qiagen). The Illumina TotalPrep-96 RNA Amplification kit was used to convert 250 ng of RNA to cRNA (Ambion, Carlsbad, CA, USA). Then, cRNA was hybridized to the Illumina Whole-Genome Gene Expression Direct Hybridization microarray (Illumina, San Diego, CA, USA). Probes that detect multiple genes were eliminated. The R software packages *lumi* and *limma* were used to calculate differentially expressed genes in treated cells. Intensity values were normalized and log-2 transformed. The Benjamini-Hochberg correction was used to correct for potential false discovery. A 1.1 fold change cutoff was then used to generate a list of responsive genes for data mining. Bioinformatic analyses of enriched gene sets were made in Pathway Studio (Ariadne Genomics, Rockville, MD). Fisher's exact test was applied to determined significantly enriched pathways. Transcription factor (TF) target enrichment, gene ontology (GO) categories, and Ariadne Pathway Categories used were provided within the software. The microarray data have been deposited with the Gene Expression Omnibus repository and will be available for public access following publication (accession number GSE51656).

### Quantitative PCR

RNA was extracted using a Qiagen RNeasy kit then reverse transcribed using SuperScript III reverse transcriptase system (Invitrogen). Quantitative PCR was then performed using Fast SYBR Green Master Mix (Applied Biosystems, Carlsbad, CA, USA) on a 7500 fast real-time PCR system (Applied Biosystems). Primers for these genes were designed using Primer BLAST (Additional File 1). Fold changes were calculated using the ΔΔCt method normalized to 36B4, a housekeeping gene (*36B4* forward, 5′-GTGTTCGACAATGGCAGCAT-3′; *36B4* reverse, 5′-GACACCCTCCAGGAAGCGA-3′).

### Western Blot Analysis

Cells were serum starved 24 hours prior to treatment and restoration to normal medium. Ligand-treated cells were lysed in RIPA lysis buffer. Protein concentrations were measured using Qubit Protein Assay Kit (Invitrogen). 50 µg of protein was loaded into standard 10% polyacrylamide gels. After protein separation, SDS-PAGE gels were transferred to PVDF membranes (Millipore, Billerican, MA, USA). Membranes were then blocked in 10% nonfat milk dissolved in TBST than probed with antibodies directed against LXRα (proprietary, C. Gabbi), LXRβ (GeneTex Cat no. 89661), Skp2 (Santa Cruz sc-7164), EGFR (Santa Cruz sc-03), phospho-EGFr (Tyr1173) (Invitrogen 18-2465), ERK1/2 (Cell Signaling 9102), phospho-ERK1/2 (Thr202/Try204) (Cell Signaling 4377),or β-actin (Sigma-Aldrich A2228) in 1% milk overnight. Membranes were then washed of unbound antibody and reprobed with secondary antibodies conjugated to horseradish peroxidase (HRP) for at least 1 hr. HRP bound antibodies were then exposed to ECL reagent (Thermo Fisher Scientific, Rockford, IL, USA), which allows for their detection by film. Purified LXRα and LXRβ, a gift of Gudrun Toresson, was generated as previously described [21]. Fold change quantification was determined by densitometric analysis available in ImageJ software (version 10.2) [22].

## Results

### Expression of LXR Isoforms in Pancreatic Cancer Cells and Clinical Samples

**Figure 1:**
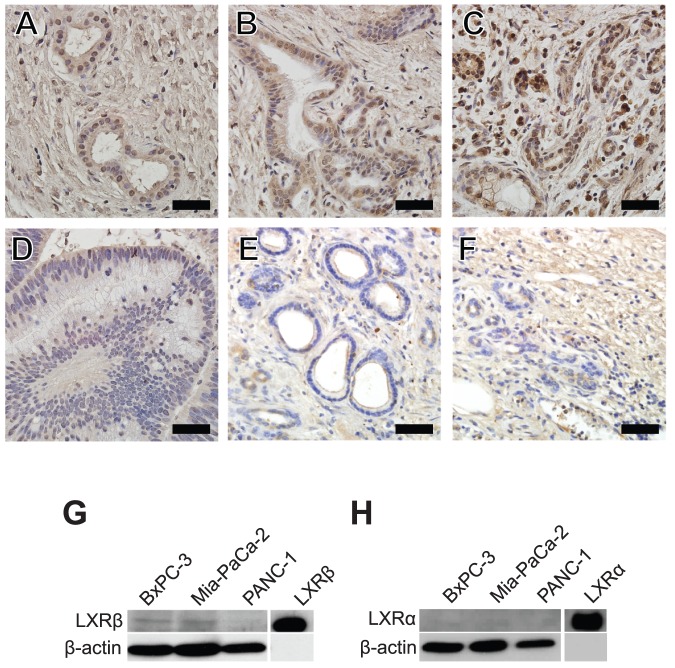
LXRβ is the main LXR isoform expressed in pancreatic cancer samples and in three pancreatic adenocarcinoma cell lines. A, LXRβ was detected in the nuclei of normal pancreatic ductal epithelial cells (female, age 59). B, C, LXRβ positive immunoreactivity was evident in both the cytosol and the nuclei of neoplastic cells of patients with pancreatic adenocarcinoma (male, age 59 and female, age 65 respectively. D, LXRβ expression was undetectable in the pancreatic adenoma sample (female, age 59). E, F LXRα immunoreactivity is not detectable in normal ductal epithelial cells (female, age 59) and in pancreatic adenocarcinoma (male age 65). G, LXRβ is expressed in BxPC-3, Mia-PaCa-2, and PANC-1 cells. H, LXRα is not expressed in PDAC cell lines. Scale bar = 50 µM.

Before characterizing the effect of LXR ligands on pancreatic cancer cell biology, which we hypothesize will restrain proliferation-related processes, we first examined LXRα and LXRβ expression in human pancreatic tumor samples and PDAC cell lines. Immunohistochemical staining of LXRβ in human samples demonstrated nuclear immunoreactivity in normal pancreatic ducts (Figure 1A). Nuclear and cytoplasmic LXRβ immunoreactivity was detected in PDAC samples (Figure 1B–C), suggesting altered localization of LXRβ in these cancerous samples. Comparatively, LXRβ expression was barely detectable in a pancreatic adenoma clinical sample (Figure 1D). Immunostaining for LXRα was not detectable both in normal ducts (Figure 1E) and in a PDAC sample (Figure 1F). These results suggest that LXRβ is the main isoform present in pancreatic ductal epithelial cells and its expression and potentially abnormal localization is evident in PDAC patient tissues.

For functional studies, BxPC-3, MIA-PaCa-2, and PANC-1 PDAC cell lines were chosen for characterization because they exhibit different invasive, proliferative, and angiogenic potential [23]. Western results indicate that LXRβ was detected in BxPC-3 and MIA-PaCa-2 and PANC-1 cells, although expression levels were the lowest in the PANC-1 cells (Figure 1G). Consistent with our observations in clinical samples, LXRα was not detected in PDAC cell lines (Figure 1H). LXR agonist GW3965 also activated expression of ABCA1, a known LXR target gene [24], in all three cell lines (Figure S1). These findings indicate that LXRβ is expressed and functional in PDAC cells

### Anti-proliferative Effects of LXR Ligands

**Figure 2:**
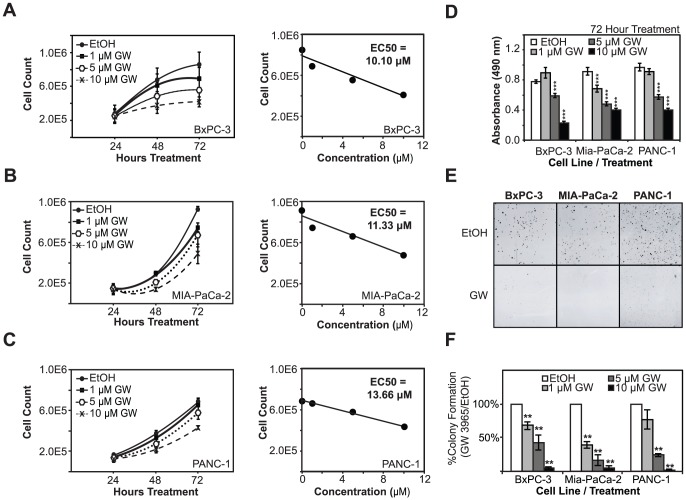
LXR agonists block cell proliferation and colony-formation in pancreatic cancer cells. A, B, C, PDAC cells (BxPC-3, Mia-PaCa-2, and PANC-1 cell lines, respectively) show dose-dependent decreases in cell proliferation upon treatment with increasing GW3965 concentrations. EC50 calculations indicate that BxPC-3 and Mia-PaCa-2 cells are more sensitive to ligand treatment than PANC-1 cells. D, Results from MTS assays, a separate measure of overall cell metabolic rate and indirect measurement of cell proliferation, demonstrate a dose-dependent drop in overall metabolism in cells treated with increasing concentrations of GW3965. E, Colony-formation ability in all three cell lines was blocked by GW3965 treatment. F, Colony formation of GW3965 treated cells was quantified relative to vehicle-treated controls. Asterisks indicated statistically significant changes.

**Figure 3:**
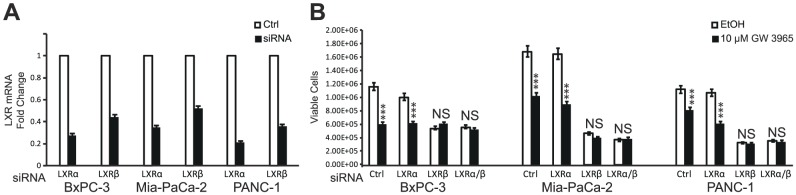
Knockdown of LXRβ expression block PDAC cell proliferation and response to LXR ligand treatment. A, Knockdown of LXRα and LXRβ expression was validated by quantitative PCR. Expression data were normalized to 36B4 ribosomal gene transcript levels. B, The effect of LXR knockdown on PDAC cell proliferation was quantified by cell counts following trypan blue exclusion assays. Asterisks indicated statistically significant changes.

To determine the effects of LXR ligands on PDAC cell proliferation, cells were treated with synthetic LXR agonist GW3965 and live cells were quantified using trypan blue exclusion assays. BxPC-3 (Figure 2A), MIA PaCa-2 (Figure 2B), and PANC-1 (Figure 2C) cell proliferation was significantly inhibited by GW3965 treatment. At 72 hours, cell numbers were significantly lower in treated cells as compared to vehicle treated controls for all three cell lines. Titration curve experiments showed a dose-dependent inhibition of cell proliferation in all three cell lines. EC50 calculations indicated that BxPC-3 and MIA-PaCa-2 exhibited the greater GW3965 sensitivity (10.10 µM in BxPC-3 and 11.33 in MIA-PaCa-2), and PANC-1 cells were the least sensitive (13.66 µM). Additional studies using tetrazolium salt reduction assays further confirmed that GW3965 suppresses the growth of PDAC cell lines in a dose-dependent manner (Figure 2D). All three cell lines showed statistically significant decreases in cell proliferation as measure by MTS reduction assays at 5 and 10 µM GW3965 for 72 hours as compared to vehicle-treated controls(***P-Val<0.001). Clonogenic assays were also employed to evaluate the effects of long-term LXR ligand treatment on cell proliferation and colony formation. Activation of LXR using GW3965 strongly inhibited colony formation in each cell line (Figure 2E–F). Inhibition was dramatic and statistically significant at 5 and 10 µM GW3965 (***P-Val<0.001 in all three cell lines). Colony formation was inhibited by over 95% in all three PDAC cell lines when treated with 10 µM GW3965 (Figure 2F). These findings suggest that LXRs are involved in PDAC cell proliferation and targeting LXRs with ligands perturb their normal functions in cell proliferation. To test this hypothesis and to determine the role of LXRs in mediating the effects of the ligands, we knocked down LXRα and LXRβ expression using small interfering RNAs (siRNAs). Transfection of PDAC cells reduced LXR expression 50–80% as compared to the controls (Figure 3A). Knockdown of LXRα had no effect on cell proliferation or response to treatment with the GW3965 ligand (Figure 3B). On the other hand, knockdown of LXRβ expression significantly reduced cell proliferation, even in vehicle treated cells, and ligand treatments following gene knockdown did not further reduce cell proliferation. These results indicate that LXRβ is required for PDAC cell proliferation and response to LXR ligand treatment and suggest that ligand treatment may disrupt its normal proliferative functions.

**Figure 4:**
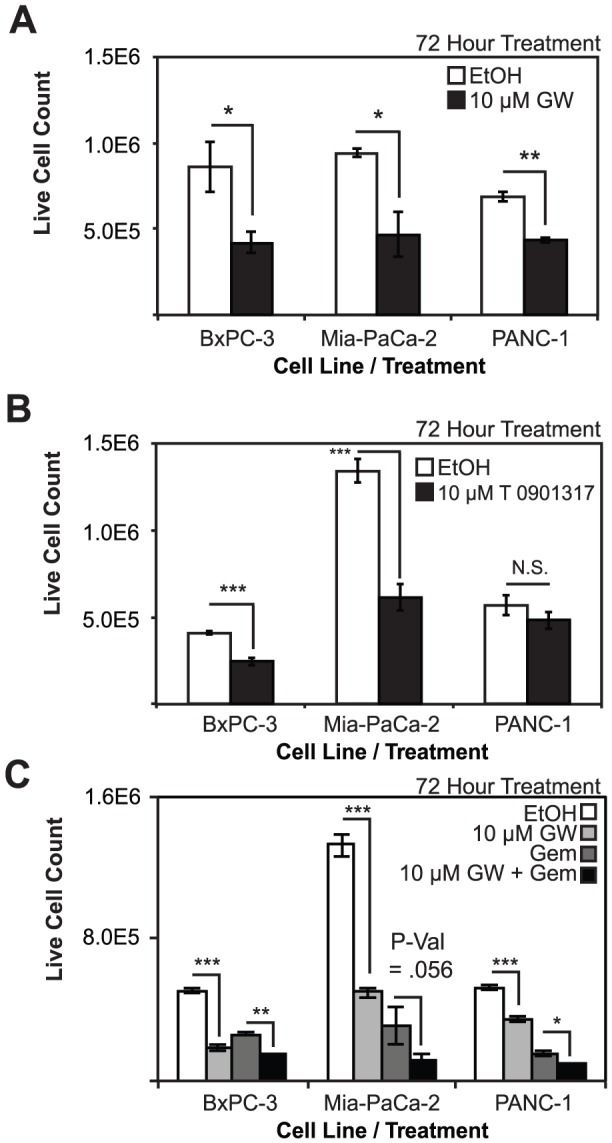
Co-treatment of pancreatic cancer cells with LXR ligands and gemcitibine reveals additive antiproliferative effects. A, Cell proliferation is blocked in BxPC-3, MIA-PaCa-2, and PANC-1 cell lines upon treatment with 10 µM GW 3965. B, LXR agonist T0901317 blocks proliferation in BxPC-3 and MIA-PaCa-2 cells, but is unable to block cell proliferation in PANC-1 cells. C, GW3965 and gemcitibine block proliferation in all three pancreatic cancer cell lines and are additive in their inhibition of proliferation when administered concomitantly. Asterisks indicated statistically significant changes.

To examine whether the observed antiproliferative effects were due to the specific synthetic agonist used in previous studies, we treated PDAC cells alternatively with the T0901317 ligand. Treatments with T0901317 blocked proliferation in two cell lines, BxPC-3 and Mia-PaCa-2, but not PANC-1 (Figure 4A). T0901317 inhibited BxPC-3 and MIA-PaCa-2 proliferation by 40.2% and 54.2%, respectively, when compared to vehicle, and the differences are statistically significant (***P-Val<0.001). PANC-1 cell proliferation was inhibited 15.3%, but the effects were not statistically significant. To mitigate potential off-target effects posed by higher ligand doses, we treated PDAC cells at a titration of lower concentrations for longer time periods. Lower concentrations of ligands elicited reproducible anti-proliferative effects, although, expectedly, to a much lesser extent (Figure S2). That there is an effect at lower concentrations suggests, however, that at least some of the effects are due to specific actions of the ligand on LXR and not through off-target mechanisms. Despite a response by PANC-1 at significantly lower concentrations of drug, there was never the precipitous decrease in proliferation as observed in BxPC-3 and MIA-PaCa-2 at higher concentrations. A similar titration experiment was performed using T0901317. The titration curves presented with a bimodal pattern, suggesting potential off-target effects depending on the concentration of ligand used (Figure S2D–F). Similar to GW 3965, T0901317 was most effective at 10 µM concentrations in all three cell lines. These findings suggest that there are ligand- and cell type-specific effects of LXR activation in PDAC cells, and the underlying mechanisms may differ depending on the concentration of ligands used in the treatments.

After demonstrating the antiproliferative effects of LXR ligands, we then compared their effects on PDAC cells to gemcitabine, a nucleoside analog chemotherapeutic with severe side effects. Cells were treated with vehicle, GW3965, gemcitabine (20 nM for BXPC-3, and 40 nM for MIA-PaCa-2 and PANC-1 cells), or combination of GW3965 (10 µM) and gemcitabine. Interestingly, GW3965 cooperated with gemcitabine to block proliferation in three pancreatic cancer cell lines to a greater extent than any treatment by itself. As expected, gemcitabine treatments inhibited proliferation in BxPC-3 by 49%, MIA-PaCa-2 by 77%, and PANC-1 cells by 71%; and the effects are significantly different when compared to vehicle (***P-Val<0.001) (Figure 4C). Co-administration of GW3965 and gemcitabine blocked proliferation in BxPC-3, MIA-PaCa-2, and PANC-1 cells by an additional 21.8%, 13.9%, and 10.5% respectively when compared to gemcitabine alone (*P-Val<0.05 in BxPC-3 and PANC-1 cells, P-Val = 0.056 in MIA-PaCa-2 cells). At a lower concentration (1 µM) of GW3965, combined treatments with different concentrations of gemcitabine, showed no additive effects, with the exception of minimal but reproducible effects with 1 nM of gemcitabine (Figure S3).

### Effects of Ligand Treatment on Cell Cycle Progression

**Figure 5:**
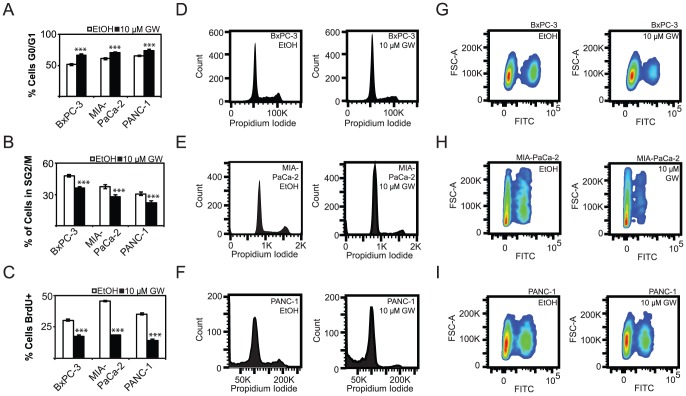
LXR agonists block pancreatic cancer cell progression through the cell cycle. A, GW3965 treatment arrests a significant proportion of the cells in the G1/G0 stage of the cell cycle as measured by propidium iodide staining and flow cytometry. B, Fewer cells are found in S, G2, or M phases following ligand treatment. C, BrdU-pulse analysis demonstrates that GW3965 treatments reduce transit through the S-phase of the cell cycle. D, E, F Representative cell cycle analysis diagram of BxPC-3, MIA-PaCa-2, and PANC-1 cells respectively. G, H, I Density plot depicting the number of cells staining for BrdU as a measure of S-phase transit in BxPC-3, MIA-PaCa-2, and PANC-1 cells. Asterisks indicated statistically significant changes.

**Figure 6:**
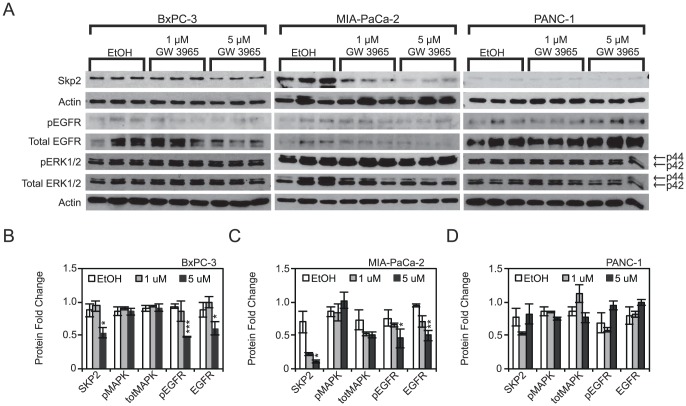
GW 3965 downregulates oncogenes involved in cancer progression. A, GW3965 treatment downregulates SKP2 and EGFR protein levels in BxPC-3 and MIA-PaCa-2 cells. Downregulation of EGFR was concomitant with a downregulation of its own phosphorylation in BxPC-3 and MIA-PaCa-2 at 5 uM GW 3965. ERK1/2 and its phosphorylation were not statistically different in any of the cell lines B, C, D Densitometric quantification of SKP2, EGFR, Phospho-EGFR, ERK1/2, and Phospho-ERK1/2 upon treatment with GW3965. Samples were normalized to actin controls. Asterisks indicated statistically significant changes.

Functional assays revealed that LXR ligand treatment blocked proliferation of PDAC cells. To better understand the mechanics of the antiproliferative effect, cell cycle analysis was performed following agonist treatment. Flow cytometry analysis revealed an additional 15.0% of BxPC-3 cells, 9.6% of MIA-PaCa-2 cells, and 8.4% of PANC-1 cells in G1/G0 phases of the cell cycle when treated with GW3965 (Figure 5A), and a corresponding 12.0% decrease of BxPC-3 cells, 9.9% of MIA-PaCa-2 cells, and 9.0% of PANC-1 in cells in S/G2/M phases of the cell cycle (Figure 5B). These changes are statistically significant (P-Val<0.001). Bromodeoxyuridine (BrdU) incorporation experiments showed a decrease in DNA synthesis by 12.9%, 27.0%, and 21.0% in BxPC-3, MIA-PaCa-2, and PANC1 cells respectively (Figure 5C) (***P-Val<0.001). Representative histograms for BxPC-3 (Figure 5D), MIA-PaCa-2 (Figure 5E), PANC-1 (Figure 5F) demonstrate a qualitative increase in G1 cells and a decrease in G2/M cells in GW 3965 treated cells. Similarly, BrdU-incorporation density plots for each cell line demonstrate a qualitative decrease in BrdU+ cells upon treatment with GW 3965 in all three cell lines (Figure 5G–I). Taken together, these findings demonstrate that LXR agonists inhibited PDAC cell proliferation by blocking cell cycle progression. To further uncover potential mechanisms of this effect on the cell cycle, we determined protein expression of cell cycle mediators known to be regulated by LXR ligand treatment in breast cancer cell lines [24]. Western analysis showed that SKP2, protein product of an oncogene, is downregulated 1.6 fold in BxPC-3 cells, 6.4 fold in MIA-PaCa-2 cells, and unchanged in PANC-1 cells when treated with 5 µM GW 3965 (Figure 6A–D) (*P-Val<0.05 in BxPC-3 and MIA-PaCa-2 cells, whereas P-Val = 0.43 in PANC-1 cells). Decreases in SKP2 were observed in MIA-PaCa-2 and PANC-1 cells following treatments with 1 µM of ligand but the changes did not reach statistical significance. A mechanism tying LXR directly to SKP2 transcriptional regulation, however, is not likely, as transcription levels do not correspond to protein levels upon treatment with GW3965 (Figure S4). This suggests that other, more upstream regulators are responsible for the observed antiproliferative effect. We specifically examined the expression of EGFR, a factor overexpressed in pancreatic cancers and the only non-chemotherapeutic marker that has been successfully targeted in the treatment of PDAC [5]. EGFR is repressed 1.45 and 1.88 fold in the more sensitive BxPC-3 and MIA-PaCa-2 cell lines upon treatment with 5 µM GW3965 (Figure 6B–C), and is statistically significant when compared to vehicle. This decrease in EGFR expression was not observed in PANC-1 cells, possibly due to their lesser sensitivity to LXR ligands (Fold Change: +1.24, P-Val = 0.19) (Figure 6D). Changes to EGFR levels were not significant following treatments with 1 µM of GW3965, although the decreasing trend is apparent in MIA-PaCa-2 cells. Decreases in EGFR expression levels in BxPc-3 and MIA-PaCa-2 coincide with decreases in phospho-EGFR (Tyr1173). Phospho-EGFR levels decrease 1.93 fold in BxPC-3 and 1.65 fold in MIA-PaCa-2 (Figure 6A–C). To further assess the downstream effects of a downregulated EGFR in BxPC-3 and MIA-PaCa-2, ERK (p44/p42) and phosphorylation status were detected. No statistically significant changes to either total ERK or phospho-ERK were observed, suggesting that a downregulated EGFR could effect change on cell proliferation through other mechanisms. These findings suggest that GW3965 inhibits transit of PDAC cells through the cell cycle, possibly by regulating key proteins that are responsible for G1-S transition and growth factor receptors that are heavily involved in regulating cell migration, proliferation, and survival [5], [25].

### Microarray Analysis of Effects of LXR Ligands on Gene Expression

**Figure 7:**
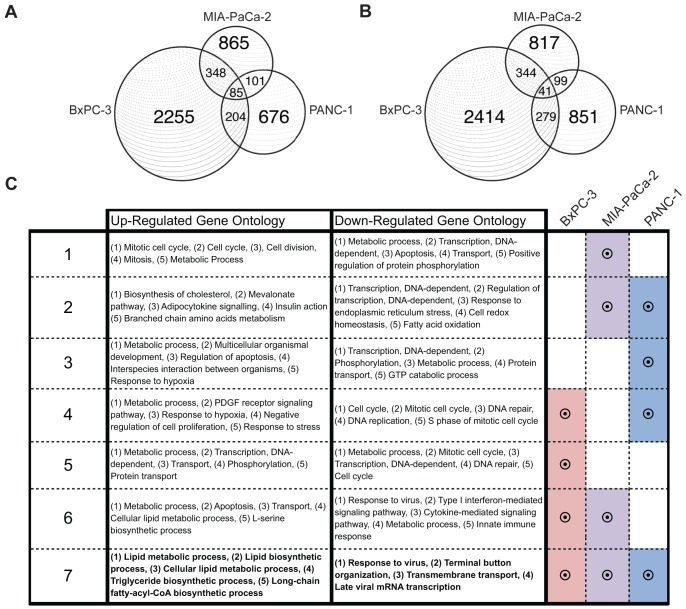
Microarray analysis of pancreatic cancer cell lines treated with LXR ligands defines common and cell line-specific effects on gene networks. A, B, Venn diagrams of up-regulated and down-regulated genes (1.1 fold change cutoff) after treatment with GW 3965 for 72 hours. These cell lines show common and cell-line specific transcriptomic responses to ligand treatment. C, Microarray analysis of up-regulated genes show that all cell lines share up-regulation of lipid metabolic, glucose metabolic, and cell proliferation responses. All cell lines down-regulate pathways that regulate response to viral infection, transmembrane support, as well as viral mRNA transcription. Treatments of BxPC-3 and PANC-1 cells down-regulate the expression of genes involved in cell cycle and DNA replication machinery.

Activation of LXR, a ligand-dependent transcription factor, is expected to directly or indirectly alter the expression of genes involved in proliferation-related pathways in pancreatic cancer cells. Microarray analysis of GW3965 responsive genes in three PDAC cell lines revealed common and cell line-specific responses. BxPC-3, MIA-PaCa-2, and PANC-1 cell lines showed distinct differences in the total number of up-regulated genes, numbering 2255, 865, and 676 in each respective cell line (Figure 7A). Of these, only 85 had concordant responses in all three cell lines. A similar distribution of down-regulated responsive genes was noted in the three cell lines, with the most robust response observed in BXPC-3 cells, with 41 genes commonly down-regulated in all three cell lines (Figure 7B). Gene ontology and pathway analysis of responsive genes showed that ligand treatment up-regulated genes involved in lipid metabolic, triglyceride biosynthetic, and long-chain fatty-acyl-CoA biosynthetic processes, including previously identified LXR target genes (Figure 7C). This is consistent with LXR's known roles in cholesterol and lipid metabolism in other tissues [24]. Commonly down-regulated genes include those that regulate cellular response to viral infection (Figure 7C). Down-regulated pathways that were shared between BxPC-3 and PANC-1 cell lines regulate cell cycle progression and DNA replication, while down-regulated pathways shared between BxPC-3 and MIA-PaCa-2 regulate modulators of immune response, such as the innate immune response and type I interferon-mediated pathways (Figure S4). Pathways responsible for cytoskeleton organization, apoptosis, and inflammatory-related pathways are also differentially expressed, which suggests that LXR ligands may regulate other cancer-related processes such as metastasis or cell survival in models of PDAC. These results indicate that activation of LXRs using LXR ligands result in dramatic antiproliferative and anticlonogenic effects in PDAC cells in general, but the underlying mechanisms of action appear to be varied.

## Discussion

In this study, we tested the hypothesis that LXR activation with synthetic agonists can halt the proliferation of pancreatic cancer cells. Before assessing the effects of LXR ligands in PDAC cells, we first demonstrated that LXRβ is the main LXR isoform expressed in human pancreatic ductal epithelial cells, as LXRα is not detectable in human normal pancreatic ducts (figure 1E), pancreatic adenocarcinoma (Figure 1F), or in PDAC cell lines (Figure 1H). Our studies uncovered variation in the expression levels of LXRβ in PDAC cell lines, as well as differences in sub-cellular localization of LXRβ in PDAC primary samples (Figure 1A–D). Unliganded LXRβ has been shown to be partially exported from the nucleus to the cytoplasm [26], suggesting that there are either differences in endogenous activating ligands in clinical samples, or variable regulation of mechanisms involved in nuclear import/export where cytoplasmic staining of LXRβ is stronger (Figure 1B–C). Differential localization of LXRβ proteins in clinical samples suggests that LXRβ may be suppressed in malignant cells by exclusion from the nucleus, but a more comprehensive study is needed to determine whether cytoplasmic staining of LXRβ is associated with disease progression and patient survival.

Functional assays clearly demonstrated that activation of LXRs by GW3965 in PDAC cell lines resulted in dramatic decreases in proliferation as measured by trypan blue exclusion assays (Figure 2A–C). Calculations of the EC50 for individual cell lines revealed that BxPC-3 and MIA-PaCa-2 cells were more sensitive to ligand treatment than PANC-1 cells. This difference in response may be due to the lower expression of LXRβ in this cell line when compared to others (Figure 1E). PANC-1, therefore, may represent a more refractory cell line. This notion is further supported by clonogenic assays, which demonstrate reduced PANC-1 sensitivity to ligand treatment (Figure 2F), as well as complete PANC-1 insensitivity to another LXR agonist T0901317 (Figure 4B). T0901317 is a promiscuous binder of other nuclear receptors, such as farnesoid X and RAR-related receptors, which may explain why the effects of GW3965 (Figure 4A) are not completely recapitulated by T0901317 [9], [27], [28], [29]. The diverging effects of alternative LXR ligands may also be attributable to differences in LXRβ expression levels, metabolism of the compound, or epigenetic modifications that potentiate alternative mechanisms. Recent advances in the treatment of PDAC pair the existing pair standard-of-care chemotherapeutic gemcitabine with an EGFR tyrosine kinase inhibitor such as erlotinib. Before this, gemcitibine was the first therapeutic that was able to extend survival since it replaced 5-fluorouracil as the preferred chemotherapeutic agent in 1997 [30]. This suggests that combination therapy including gemcitabine is one of a limited set of viable strategies in the development of therapeutics for pancreatic cancer. We demonstrate here that gemcitabine concomitant with GW3965 may be superior to either treatment by itself in the three pancreatic cancer cell lines (Figure 4C).

In functional studies to determine the role of LXR in mediating the effects of ligands, LXRβ knockdown led to a dramatic decrease in the proliferation of PDAC cells (Figure 3B). This observation seemingly conflicts with the notion that LXRβ has an antiproliferative role. One possible explanation is that synthetic ligands function as agonists or activators for genes and cellular functions associated with cholesterol transport and metabolism but as antagonists of LXR regulation of genes and processes involved in cell proliferation. Another possibility is that treatments with synthetic ligands, particularly at higher concentrations, may activate negative feedback mechanisms which then lead to the inactivation or degradation of LXR, reminiscent of high doses of estrogens used to block estrogen receptor functions in the early days of endocrine treatment of breast cancer [31], [32]. Finally, the anti-proliferative actions of LXR ligands may be due to off-target effects, especially at higher concentrations, and independent of LXR activity, but the results from the studies using lower concentrations of ligands (see Figure S2) suggest that at least some of the anti-proliferative effects are mediated by LXR. These hypotheses regarding the mechanisms of action of LXR ligands and the role of LXR in pancreatic cancer await further testing in future studies.

To understand the cellular mechanisms underlying cancer cell proliferation inhibition by LXR agonists, we utilized flow cytometry to quantify changes in the cell cycle in PDAC cells. GW3965 treatment arrested PDAC cells in the G1/G0 phase of the cell cycle (Figure 5A,B). It also strongly inhibited BrdU incorporation, a measure of cell S-phase transition (Figure 5C). These data show that LXR activation by synthetic agonists results in cell cycle arrest, but does not indicate mechanisms linking LXR's known role as a transcription factor to its antiproliferative effect. Here we show that Skp2, an oncogene previously shown to be down-regulated in ligand treated breast cancer cells, is down-regulated in two sensitive PDAC cell lines as a consequence of GW3965 treatment (Figure 6B–C). Skp2 is known to regulate c-Myc transactivation and ubiquitination, and regulates the turnover of other cell cycle regulatory units in maintenance of normal G1-S transition [28], [29]. LXR ligand treatment also down-regulates epidermal growth factor receptor (EGFR) in two cell lines (Figure 5B–C), raising questions about LXRs and their effects on apoptosis and migration in PDAC, as EGFR is integrally linked to these pathways. Interestingly, activation of LXR using GW3965 has been shown to sensitize glioblastoma cells expressing EGFR splice variant (EGFRvIII) to apoptosis in *in vivo* models of glioblastoma [33]. We did not observe increases in cell death, however, following ligand treatment (Figure S5). These results suggest that LXRs are integrally tied to machinery regulating cell cycle progression and growth factor signaling.

It has been posited that gene networks involved in cholesterol and fatty-acid metabolism are tied to LXR's emerging roles in cancer cell growth [24], [34]. Activation of LXR leads to strong up-regulation of SREBF1 (sterol regulatory element-binding protein 1c) in breast, colon, pancreatic cancer cells (Figure 7C, S1), and is a regulator of lipogenesis and glucose metabolism [15], [35]. Knockdown of SREBF1 protein in breast cancer cells, however, did not block cell proliferation inhibition by LXR agonists [24]. Interestingly, published studies linked sterol metabolic pathways to proliferation of T-cells through ABCG1 in normal T cell physiology. Inactivation of this transporter prevented LXRβ-mediated inhibition of proliferation in T cells [34]. The addition of low-density lipoproteins to the medium of T cells did not interfere with cell proliferation. These findings suggest that cholesterol may not only be a constituent of the cell membrane, but may be dynamically regulated intracellularly as a component of cell cycle progression controlled by cholesterol transporters and other factors. Despite promising potential leads into mechanisms potentially regulated by LXR agonists, more work needs to be done in pancreatic tissues to elucidate how this effect is achieved.

Mechanistically, our microarray study showed both concordant and discordant gene responses in three PDAC cell lines. Up-regulated genes (Figure 7A) are enriched for those known to function in cholesterol and fatty acid metabolism (Figure 7C), whereas down-regulated genes (Figure 7B) were less concordant, and function in pathways that regulate response to viral infection (Figure 7C). Differences between cell lines in these responses can be attributed to variation in epigenetic modifications that potentiate LXR activity at response elements after ligand stimulation. Genome-wide microarray studies in breast cancer cells show that up-regulated genes tend to regulate cholesterol and fatty acid metabolism, whereas down-regulated genes function in DNA replication and cell cycle programs. Specifically, treatments with LXR agonists down-regulated the expression of E2F2, a member of the E2F family of transcription factors. However, knockdown of E2F2 in breast cancer cell lines only blocked proliferation in ER+ cell lines which suggests that mechanisms of cell proliferation inhibition by LXRs may be diverse in nature [16]. It is important to note that E2F2 is repressed significantly in BxPC-3 cells and not in the other two PDAC cells, and this observation suggests that mechanisms discovered in breast cancer cells may not necessarily be involved in pancreatic cancer cells. Differences in experimental design may also explain the variations noted between tissues (i.e. breast vs. pancreas). Treatment time was 72 hours for PDAC cell lines, whereas a shorter 48 hour-treatment was used for breast cell lines. Therefore, analyses in PDAC cells likely uncovered more secondary and tertiary responses to ligand treatment, and justifiably so because inhibition of cell proliferation by LXR is not thought to be a primary response. Future studies using shorter treatment times will shed light on early mechanisms underlying the effects of LXR agonists.

These initial studies demonstrated the effects of LXR ligands on cell proliferation, but more work is needed to characterize their effects on other cancer-related processes. Treatment with LXR agonists induced apoptosis in prostate cancer cell lines and xenograft models by down-regulating AKT signaling [36]. However, our data show that ligand treatments and LXR activation in pancreatic cancer cells was solely anti-proliferative, and lacked the ability to induce apoptosis as measured by caspase-3 cleavage (Figure S5). Additional work is needed to characterize LXR function in the context of cell motility, migration, and the unique effects of other LXR ligands on pancreatic cancer biology in both cell-based and animal models. These findings, however, indicate that LXR agonists and their derivatives warrant further study and development as potential therapeutic agents in the treatment of pancreatic cancer.

## Supporting Information

Figure S1 (EPS)

Figure S2 (EPS)

Figure S3 (EPS)

Figure S4 (EPS)

Figure S5 (EPS)
